# Insights Into Potato Spindle Tuber Viroid Quasi-Species From Infection to Disease

**DOI:** 10.3389/fmicb.2020.01235

**Published:** 2020-07-03

**Authors:** Charith Raj Adkar-Purushothama, François Bolduc, Pierrick Bru, Jean-Pierre Perreault

**Affiliations:** RNA Group/Groupe ARN, Département de Biochimie, Faculté de Médecine des Sciences de la Santé, Pavillon de Recherche Appliquée au Cancer, Université de Sherbrooke, Sherbrooke, QC, Canada

**Keywords:** viroids, quasi-species, high-throughput sequencing, PSTVd, population dynamics, circular RNA, long non-coding RNAs

## Abstract

Viroids are non-coding RNA plant pathogens that are characterized by their possession of a high mutation level. Although the sequence heterogeneity in viroid infected plants is well understood, shifts in viroid population dynamics due to mutations over the course of infection remain poorly understood. In this study, the ten most abundant sequence variants of potato spindle tuber viroid RG1 (PSTVd) expressed at different time intervals in PSTVd infected tomato plants were identified by high-throughput sequencing. The sequence variants, forming a quasi-species, were subjected to both the identification of the regions favoring mutations and the effect of the mutations on viroid secondary structure and viroid derived small RNAs (vd-sRNA). At week 1 of PSTVd infection, 25% of the sequence variants were similar to the “master” sequence (i.e., the sequence used for inoculation). The frequency of the master sequence within the population increased to 70% at week 2 after PSTVd infection, and then stabilized for the rest of the disease cycle (i.e., weeks 3 and 4). While some sequence variants were abundant at week 1 after PSTVd infection, they tended to decrease in frequency over time. For example, the variants with insertions at positions 253 or 254, positions that could affect the Loop E as well as the metastable hairpin I structure that has been shown important during replication and viroid infectivity, resulted in decreased frequency. Data obtained by *in silico* analysis of the viroid derived small RNAs (vd-sRNA) was also analyzed. A few mutants had the potential of positively affecting the viroid’s accumulation by inducing the RNA silencing of the host’s defense related genes. Variants with mutations that could negatively affect viroid abundance were also identified because their derived vd-sRNA were no longer capable of targeting any host mRNA or of changing its target sequence from a host defense gene to some other non-important host gene. Together, these findings open avenues into understanding the biological role of sequence variants, this viroid’s interaction with host components, stable and metastable structures generated by mutants during the course of infection, and the influence of sequence variants on stabilizing viroid population dynamics.

## Introduction

Viroids are plant pathogenic single-stranded, circular, non-coding RNA molecules composed of 246–401 nt (Ding, 2009). Since viroids are not known to code for any peptides, they rely entirely on their sequence, structure and host factors for their replication and propagation (Flores et al., 2005). Their genome possesses sufficient sequential and structural information to take over both the plant’s defense system and its transcriptional machinery to reproduce and spread throughout the host. Upon infection, viroids induce a wide array of symptoms depending on the host plant. However, the degree of viroid induced symptoms depends on the viroid variant and the host cultivar’s susceptibility (Owens et al., 2012). To date, 32 viroid species have been classified based on the presence or the absence of the Central Conserved Region (CCR) domain that classifies viroids into two families, the *Pospiviroidae and* the *Avsunviroidae*. The members of the family *Pospiviroidae* (type species: *Potato spindle tuber viroid*; PSTVd) have five structural/function domains such as the Terminal Left (TL), the Pathogenicity (P), the Central (C), the Variable (V), and the Terminal Right (TR) domains. They replicate in the host’s nucleus in an asymmetric rolling circle mechanism. Conversely, the members of the family *Avsunviroidae* (type species: *Avocado sunblotch viroid*; ASBVd) lack the CCR, but exhibit self-cleavage via a *cis*-acting hammerhead sequence activity. These members replicate in the chloroplast of the host in a symmetric rolling circle mechanism (Adkar-Purushothama and Perreault, 2020).

Since viroids are non-coding RNA pathogens, they recruit host DNA dependent RNA polymerase during replication. Specifically, members of the family *Avsunviroidae* use nuclear-encoded polymerase (NEP), whereas the members of family *Pospiviroidae* use DNA dependent RNA polymerase II (Flores et al., 2011). Under normal conditions, the NEP and the DNA dependent RNA polymerase II use DNA as template to generate RNA molecules. Viroids redirect the NEP and the DNA dependent RNA polymerases to use the viroid’s RNA as template instead of DNA. As a consequence, the replication becomes error prone (Flores et al., 2005). Analysis of chrysanthemum chlorotic mottle viroid (CChMVd) sequences retrieved from the plant infected with a single variant of CChMVd, revealed a mean error rate of 2.5 × 10^–3^ per nucleotide position. In other words, the 399 nt long CChMVd had one error for every 400 nt, the highest reported mutation rate for any given biological species (Gago et al., 2009). High-throughput sequence analysis of peach latent mosaic viroid (PLMVd) recovered from the peach tree inoculated with a single variant of PLMVd revealed the presence of 3,939 sequence variants (Glouzon et al., 2014). The data showed that most of the sequence variants had an average of 4.6–6.4 mutations as compared to the initially inoculated, or master, sequence. On the other hand, analysis of PSTVd-derived small RNA (PSTVd-sRNA) by deep-sequencing of PSTVd-infected plants revealed that the mean error rate per nucleotide was less than 5 × 10^–3^, which lies within the range observed for members of the family *Avsunviroidae* (Brass et al., 2017). Additionally, analysis of the mutation rate for the chloroplast replicating viroid, eggplant latent viroid (ELVd) and nuclear replicating PSTVd in a common host revealed higher mutation frequencies in ELVd than in PSTVd (López-Carrasco et al., 2017). The sequence variants created by the replication of a master sequence during the course of replication are called a “quasi-species” or “viroid cloud” or “mutant swarm” (Eigen, 1971; Domingo, 2002; Glouzon et al., 2014; Domingo and Perales, 2019).

Viroids, as a minimal pathogen consisting of a naked circular single-stranded RNA molecule, are dependent on their thermodynamically stable structure and nucleotide sequence for their survival. Viroids rely on both thermodynamically stable, as well as metastable, secondary structures to interact with host components for their biological functions (Repsilber et al., 1999; Flores et al., 2012). Recently, direct visualization of the native structure of PLMVd at a single-molecule resolution using atomic force microscopy confirmed the stabilizing role of tertiary structures such as kissing-loop interactions (Moreno et al., 2019). Furthermore, it has been demonstrated that the kinetically preferred metastable structure containing hairpin I (HPI) and hairpin II (HPII) of PSTVd is crucial for both its replication and infectivity (Hammond and Owens, 1987; Loss et al., 1991; Gas et al., 2007). However, studies have also shown that certain changes in the nucleotide sequence of the viroid’s RNA can be associated with the disease severity (Rodriguez and Randles, 1993; Semancik and Szychowski, 1994; De la Peña et al., 1999; Schnell et al., 2001; De la Peña and Flores, 2002; Malfitano et al., 2003; Maniataki et al., 2003; Tsushima et al., 2016; for a review see Adkar-Purushothama and Perreault, 2020). These findings, along with the detection of viroid-derived small RNAs (vd-sRNAs) in the PSTVd infected plants implicated RNA interference (RNAi, also known as RNA silencing) in viroid pathogenicity (Itaya et al., 2001; Papaefthimiou et al., 2001; Martinez de Alba et al., 2002). Due to their highly base-paired secondary structures, viroids trigger the host’s RNA silencing machinery (Pallas et al., 2012). Although the vd-sRNAs are active in guiding the RNA-induced silencing complex (RISC)-mediated cleavage, mature PSTVd is partly resistant to the RISC-mediated cleavage due to its secondary structure (Itaya et al., 2007; Sano et al., 2010; Adkar-Purushothama et al., 2015b; Dalakouras et al., 2015). Several studies using various viroid–host combinations demonstrated the down-regulation of the host’s mRNA by its direct interaction with the vd-sRNA (Navarro et al., 2012; Eamens et al., 2014; Adkar-Purushothama et al., 2015a, 2017; Zheng et al., 2017; Adkar-Purushothama and Perreault, 2018). Previously, it has been shown that changes in a few critical nucleotides in the seed region of the vd-sRNA/mRNA complex has substantial effects on the efficiency of RNA silencing of the target mRNA (Adkar-Purushothama et al., 2015a). For example, changing two nucleotides of the seed region of the vd-sRNA of PSTVd-I targeting the *callose synthase 11-like (Cals11-like)* mRNA to that of PSTVd-M negatively affected the down-regulation of *Cals11-like* mRNA and, consequently, the viroid induced symptoms (Adkar-Purushothama et al., 2015a). Recently, analysis of polysome fractions of viroid infected plants revealed the direct interaction of viroid and vd-sRNA with the host’s translation machinery, thus inducing ribosomal stress in the host plant (Cottilli et al., 2019). However, given the fact that viroids are quasi-species, it is not clear whether or not the interaction detected with the host translation machinery is specific to the viroid’s structure or it’s sequence. Hence, it is crucial to understand the specific structures and sequence variants of a viroid that are involved in the different stages of infection.

Previously it has been noted that PSTVd follows the S-curve in diseased plants. More specifically, after inoculation, PSTVd RNA showed a brief lag phase that was followed by a sudden increase in its titer before the stationary phase and finally a drop in its titer (Adkar-Purushothama and Perreault, 2018). As viroids depend solely on their structure and their sequence for establishing themselves and for conquering the plant’s defense mechanism, a shift in viroid sequence dynamics during the course of infection was suspected. To date, different approaches have been used to study viroid’s quasi-species nature in viroid infected plants (Gago et al., 2009; Glouzon et al., 2014; Brass et al., 2017; López-Carrasco et al., 2017). However, all of these experiments were restricted to a single point sample collection, and much of the work was focused on estimating the mutation rate. Hence, in this present study, samples of PSTVd infected tomato plants were collected at different time intervals and subjected to deep-sequencing and computational analysis to understand: (i) the evolution of the viroid quasi-species during the course of infection; (ii) the effect of these mutations on stable secondary structures; and, (iii) any changes in their vd-sRNA – host target specificity. The findings provide more insights on how the composition of a viroid quasi-species of sequences changes during the course of infection.

## Materials and Methods

### PSTVd Constructs and Bioassays

The dimeric construct of PSTVd-RG1 (GenBank Acc. No. U23058) was previously inserted in the pBluescript KS + vector (Stratagene) and was then ligated into the binary vector pBIN61 (Adkar-Purushothama et al., 2017). Briefly, the dimeric PSTVd-RG1 construct was purified after being subjected to digestion with the restriction endonucleases *Xba*I and *Bam*HI and was then ligated into the same sites of the binary vector pBIN61. The resulting recombinant binary vector was then transformed into the *Agrobacterium tumefaciens* (*A. tumefaciens*) strain GV3101 as previously described (Adkar-Purushothama et al., 2017).

Tomato seedlings (*Solanum lycopersicum* cv Rutgers; Livingston Seed Co.) were used for the bioassays. All plants were grown in a chamber at 25°C with 16 h light and 8 h darkness. The primary leaves were agro-infiltrated with the *A. tumefaciens* strain GV3101 containing the dimeric construct of PSTVd-RG1 in the binary vector pBIN61 whereas empty vector was used for mock infection. Since, *A. tumefaciens* strain GV3101 and binary vector pBIN61 are not known to move systemically, the agro-infiltrated leaves were excised at 3 days of post-inoculation to avoid continuous generation of master sequence from the vector. Upper non-inoculated, fully opened single whole leaf samples were collected at 1, 2, 3, and 4 weeks post-inoculation (wpi) from the same 3 plants for RNA preparation. In other words, every week the next youngest leaf above the previously sampled leaf was collected for RNA extraction.

### RNA Preparation, RT-PCR, and RNA Gel Blots

Total RNA from infected leaf samples was extracted using the mirVana miRNA isolation kit (Ambion) as described previously (Adkar-Purushothama et al., 2018). Briefly, 150 mg of leaf sample was ground with 600 μL of lysis/binding buffer in the presence of sand. Then, 70 μL of microRNA homogenate was added and the sample was mixed by vortexing followed by incubation on ice for 10 min. Total RNA was isolated by acid phenol-chloroform (5:1) extraction and was precipitated by centrifugation after adding 2.5 volumes (vol.) of absolute ethanol. The RNA was further purified by DNase I (Promega) treatment. RNA integrity was examined in a 2100 Bioanalyzer (Agilent Technologies). RNA obtained for each sample was analyzed by RT-PCR assay to detect viroid presence (Adkar-Purushothama et al., 2015a). Equal amounts of RNA obtained for each week were pooled together for the RNA gel blot assay and the cDNA library preparation.

To detect PSTVd, dimeric (−) PSTVd-RG1 riboprobes were prepared as described previously (Adkar-Purushothama and Perreault, 2018). For the RNA gel blot hybridizations, 500 ng of the total RNA samples pooled for each week were denatured at 65°C for 15 min with 3 vol. of sample buffer [50% formamide, 2.2 M formaldehyde (37%), and 1x MOPS], and were then separated by electrophoresis on 1.0% agarose-formaldehyde gels containing 1x MOPS buffer. The RNAs were transferred to Hybond-XL nylon membrane (Amersham, GE Healthcare Life Sciences) and hybridized with radiolabeled probes as described before (Adkar-Purushothama et al., 2018). Radiolabeled DNA probes specific for 5S rRNA used to evaluate the expression level of 5S rRNA, which was used as the loading control for the RNA gel blot assay (Adkar-Purushothama and Perreault, 2018).

### Viroid Library Preparation and High-Throughput Sequencing

For the viroid library preparation, circular PSTVd molecules were purified from the pooled total RNA of each week by separating 10 μg of total RNA by 5% polyacrylamide-8 M urea denaturating gel electrophoresis (5%-dPAGE). The circular PSTVd (cPSTVd) molecules were eluted from the gel and precipitated with 2.5 vol. of ethanol. To prepare the two complementary DNA (cDNA) libraries, 1 μg of purified cPSTVd RNA was separately subjected to reverse transcription (RT) using a primer binding at either position 280–269 (L1) or 10–354 (L2) of PSTVd-RG1, respectively, for each week. The resulting cDNA libraries were amplified by polymerase chain reaction (PCR) with Q5 High-fidelity DNA polymerase enzyme (New England Biolabs) in the presence of primers F1/R1 for L1 and F2/R2 for L2 followed by indexing of each library. The 8 libraries (2 libraries per week × 4 weeks) were sequenced using the Illumina MiSeq sequencer at the Laboratoire de Génomique Fonctionnelle de l’Université de Sherbrooke^1^. All the primers used in this study are listed in Supplementary Table 1. The deep sequence data generated in this study is deposited in the Gene Expression Omnibus under accession number GSE147577.

### Bioinformatic Analysis

**FIGURE 1:**
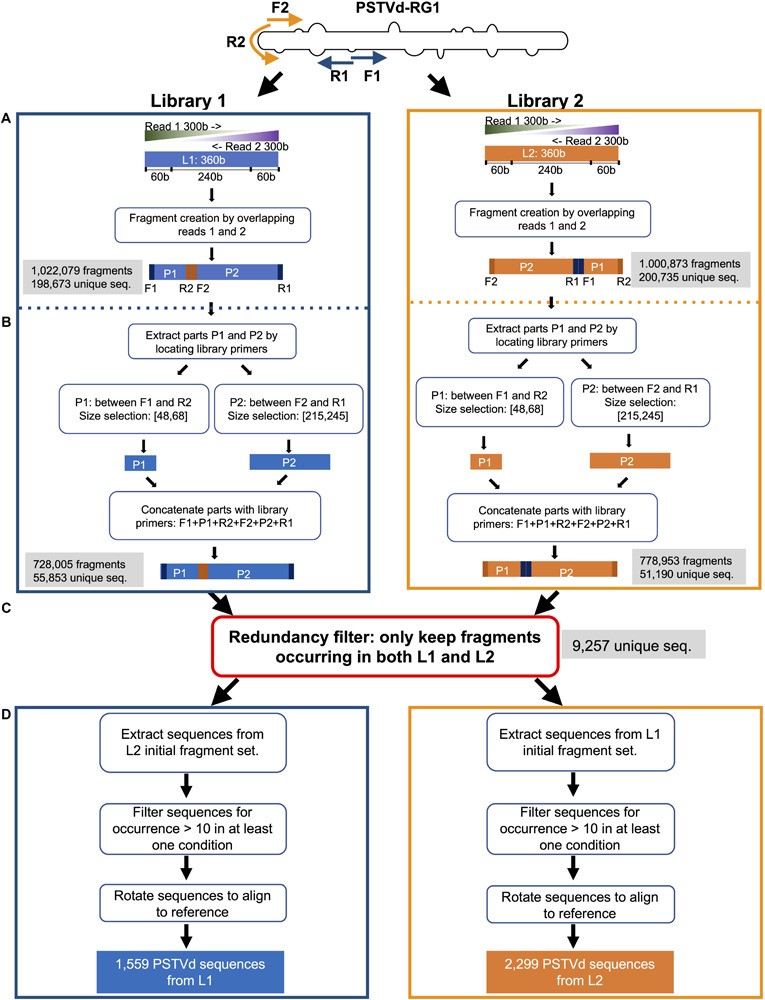
Scheme of the steps followed for the treatment of the high-throughput sequence data. **(A)** Library fragments were created by aligning read pairs by their overlapping sequences after trimming of the sequence adapters and were then filtered using sequence quality metrics. **(B,C)** The resulting sequences were extracted and evaluated for sequence redundancy between libraries 1 (L1) and 2 (L2). **(D)** The redundant sequences were extracted, and those that occurred at least ten times were filtered and aligned against the master sequence (PSTVd-RG1). In the figure, F1 and R1 indicate the forward and reverse primer used for the preparation of L1, as well as their respective binding sites on the PSTVd molecule; F2 and R2 denotes the forward and reverse primer used for the preparation of L2, as well as their respective binding site on the PSTVd molecule; P1 denotes the region located between F1 and R2; P2 denotes the region located between F2 and R1.

Workflow was divided four main steps, specifically steps A–D (Figure 1). Each step was performed independently for both libraries except for step C. All steps were implemented using a combination of Mothur commands and in-house Perl programming (Schloss et al., 2009; Kozich et al., 2013). Step A consisted of creating the library fragments by aligning the read pair in their overlapping parts. Initially, the adapter sequence were trimmed using the Trimmomatic tools (Bolger et al., 2014) and the resulting fragments were filtered against sequence quality metrics, overlapping ambiguities and length criteria. Step B consisted of removing the sequence parts that were targeted by the PCR primers for amplification to mask the PCR primer binding regions before evaluating any redundancy between the two libraries. Step C was the evaluation of the redundancy between the fragments of both libraries. A sequence was retained if it was present in both libraries. As described in step B above, the PCR primer regions were not considered in step C. Finally, step D included three sub-steps: (i) the extraction; (ii) the occurrence filtering; and, (iii) the rotation. Based on the redundancy set obtained in step C, all of the sequences that were present in both the libraries were extracted. Thus, the extracted repetitive sequences were filtered to obtain those sequences that were found at least 10 times in one of the libraries. Then, these sequences were aligned against the master sequence to identify the sequence variation.

## Results

### Infection Assays and High-Throughput Sequencing of the PSTVd Quasi-Species

**FIGURE 2:**
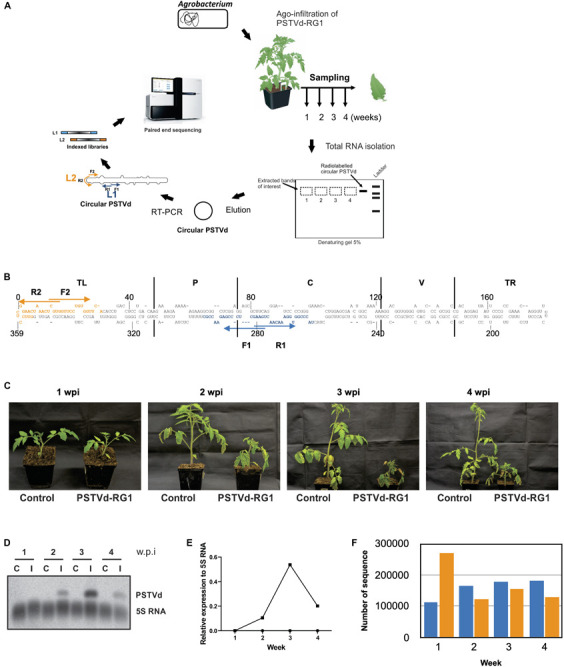
Experimental strategy and analysis of the viroids recovered from the PSTVd-RG1 infected tomato plants. **(A)** Flow diagram outlining the steps involved in the infection assay, sample preparation and viroid high-throughput sequencing from the PSTVd-RG1 infection in tomato plants. *Agrobacterium tumefaciens* (*A. tumefaciens*) strain GV3101 was transformed with recombinant binary vector pBIN61 containing the dimeric construct of PSTVd-RG1. Primary leaves of tomato plants (cv. Rutgers) which were maintained in a chamber were agro-infiltrated with the transformed *A. tumefaciens*. Plants inoculated with empty vector were used for mock infection. Total RNA was extracted from the upper, non-inoculated leaf samples which were harvested at 1–4- weeks post-infection (wpi). After verifying the presence of PSTVd, circular PSTVd (cPSTVd) was isolated through polyacrylamide gel electrophoresis under denaturing conditions. Purified cPSTVd RNA was amplified by RT-PCR using two distinct sets of primers binding at different locations on the PSTVd molecule to construct two libraries namely, Library 1 (L1) and Library 2 (L2). The resulting 8 libraries (2 libraries per week × 4 weeks) were indexed and sequenced using the Illumina MiSeq sequencer. **(B)** Nucleotide sequence and secondary structure of PSTVd-RG1. The region spanning the primers used for amplification are shown in blue and orange, respectively, for libraries 1 and 2 (i.e., L1 and L2). The arrows indicate the primer binding sites in the 5′–3′ orientation. The structural/functional domains of PSTVd: Terminal left (TL), Pathogenicity (P), Central (C), Variable (V), and Terminal right (TR), are delimited by the vertical solid lines and are named accordingly. **(C)** Photos of tomato plants agroinfiltrated at the two leaf stage with *Agrobacterium* harboring, or not (control), a plasmid including a dimeric head-to-tail construct of PSTVd-RG1. **(D)** Autoradiogram of a RNA-gel blot hybridization for the detection of PSTVd and the 5S RNA (as loading control) in PSTVd-infected tomato plants. **(E)** Relative expression level of PSTVd compared to that of 5S rRNA. **(F)** Histogram of the total number of full-length PSTVd reads obtained by high-throughput sequencing for both the L1 (blue color) and the L2 (orange color) libraries.

To assess the evolution of a viroid during the different stages of infection in the host plant, PSTVd-RG1 and tomato plants were used as a model system as this viroid-host interaction is well studied (Adkar-Purushothama et al., 2017; Adkar-Purushothama and Perreault, 2018). The flow chart outlining the steps used to create the viroid libraries is shown in Figure 2A. Briefly, leaf samples were collected from tomato plants that were agro-infiltrated with the plasmid containing a dimeric head-to-tail construct of the PSTVd-RG1 variant. At 1–4 wpi, total RNA was extracted from harvested leaves. The presence of PSTVd in agro-infiltrated plants was verified by RT-PCR, as well as by RNA gel blot assay using radiolabeled PSTVd probes. To develop a full-length PSTVd library, cPSTVd was purified from the total RNA extracted from the PSTVd infected plants by electrophoretic migration under denaturation condition and, was then amplified by RT-PCR amplification using two distinct sets of primers binding at different locations on the PSTVd molecule. Library 1 (L1) was prepared using primers binding at position 260–295 (F1/R1) of PSTVd-RG1, while library 2 (L2) was prepared using primers binding at position 354–30 (F2/R2) of PSTVd-RG1 (Figure 2B). Construction of the two libraries provided information on the entire whole viroid genome, including the primer binding sites. Illumina indexed primers were used to identify the origin of each sequence with respect to both the library and the number of weeks post-infection.

The tomato plants were agro-infiltrated with the plasmid containing a dimeric head-to-tail construct of PSTVd-RG1 (hereafter named the master sequence) in *A. tumefaciens*. Disease symptoms such as mild stunting and leaf curling appeared during the 2nd week of infection, followed by severe necrosis in week 3 (Figure 2C). In contrast, mock inoculated control tomato plants did not show any disease symptoms. The presence of PSTVd in infected plants was confirmed by RT-PCR reactions performed on RNA extracted from leaf samples at 1, 2, 3, and 4 wpi using the VidR1/VidF1 primers as described previously (Adkar-Purushothama et al., 2015a). Equal amounts of RNA from three plants were pooled for each week, and the amount of PSTVd present was analyzed by RNA gel blot assay (Figure 2D). Analysis of the accumulation of PSTVd after normalization against the 5S rRNA revealed that the PSTVd titer increased initially from week 1 to 3, and that it then decreased in week 4 (Figure 2E). This variation is in good agreement with previously published data (Adkar-Purushothama and Perreault, 2018).

To evaluate the accumulation of PSTVd-RG1 sequence variants over the time course of the infection, cPSTVd was purified by 5% polyacrylamide – 8 M urea gel electrophoresis followed by the preparation of both libraries as described above. Both libraries were paired end sequenced using an Illumina MiSeq sequencer, and 300 bp reads were filtered using bioinformatics tools (see section “Materials and Methods”). Thus, the sequence of each variant, as well as the number of times it was sequenced, was known for each week. In the analysis of the data, the sequence variants that were detected at least ten times in both libraries for a given week were retained. In the case of L1, 1,559 sequence variants were found at least 10 times out of 639,187 total sequences, while for library L2, 2,299 sequence variants out of 677,758 total sequences found at least 10 times. The total number of sequences retrieved in L1 and L2 for each week is presented in Figure 2F. Finally, each sequence variant was aligned to the master sequence (PSTVd-RG1). The most distant sequence variant of the L1 exhibited 95.4% identity to the master sequence, while in the case of the L2 it was 93.9%. The higher mismatch observed for L2 is due to the single sequence variant that had several mutations. Excluding this sequence variant from the analysis resulted in having almost the same sequence similarity in both the libraries.

### Mutations That Either Decrease or Increase Accumulation

To identify the different mutations that might explain the adaptation of PSTVd to its host, the sequence variants were analyzed and sorted according to their abundance each week. To reduce the number of variants to be analyzed, the ten most abundant variants for each week were retained. In addition, the proportion of each sequence variant decreases considerably beyond the ten most abundant variants, thus making the biological importance of the infrequent variants relatively insignificant. This observation is particularly true for weeks 2–4. At week 1, a greater dispersion of the variants was observed (Supplementary Figure 1). Certain sequence variants are found in the top ten for both libraries, while others are present in one. It is important to note that there is some variability in the proportions of the different variants since the primers used for the construction of two libraries do not bind to the same viroid regions. Hence, there may be different binding constraints depending on the context, that result in better or worse PCR amplification (i.e., secondary and tertiary structures, thermodynamic parameters, etc.). To ensure that real true variants that will allow drawing of solid conclusions about the adaptability of PSTVd to its host were identified, it was further decided to keep the top ten most abundant variants found in both libraries. The variants that contain mutations associated with a decrease in accumulation are listed in Table 1 while those containing mutations associated with an increase in accumulation are listed in Table 2. Analyzing the sequence variants based on their abundance through time can reveal the importance of some mutations and could explain adaptation or fitness. When analyzing the evolution of the master sequence, in either library L1 or L2, its abundance increased from 22 to 25% in week 1 and quickly reached 72–77% at week 2. Subsequently, the abundance remained steady at about 70–72% in weeks 3 and 4 (Supplementary Figure 1 and Table 1). The master sequence seems to be well adapted to its host since it represents ∼70% of the quasi-species.

**TABLE 1:** Abundance (%) of sequence variants common to both libraries for each week that bear mutations associated with a reduced accumulation.

**Variants**		**Week 1**	**Week 2**	**Week 3**	**Week 4**	Rate of decrease* (times)
Ins:C253	L1	20.1103	0.1045	0.0408	0.7745	26.0
	L2	24.7860	0.1622	0.0733	0.5159	48.0
Ins:G254	L1	5.3295	0.0224	0.0056	0.2507	21.3
	L2	8.1040	0.0468	0.0193	0.1944	41.7
Ins:U254	L1	1.3632	0.0073	0.0039	0.0487	28.0
	L2	1.4970	0.0121	0.0051	0.0333	45.0
Ins:A254	L1	1.3605	0.0091	0.0022	0.0602	27.6
	L2	2.8546	0.0202	0.0064	0.0643	44.4

*Rate of decrease: abundance at week 1/abundance at week 4.

**TABLE 2:** Abundance (%) of sequence variants common to both libraries for each week that bear mutations associated with an increased accumulation.

**Variants**		**Week 1**	**Week 2**	**Week 3**	**Week 4**	Rate of increase* (times)
Ins:A55	L1	0.5989	0.9771	1.0361	1.1314	1.9
	L2	0.5156	0.7862	0.8788	0.6088	1.2
Del:A55	L1	0.0447	0.5511	0.9814	4.2924	96.0
	L2	0.0438	0.5085	0.8994	4.1564	94.9
Sub:G168A	L1	0.1189	0.3843	0.2864	0.2567	2.2
	L2	0.1148	0.2978	0.2957	0.2603	2.3
Del:U296	L1	0.2128	0.6236	0.6481	0.5994	2.8
	L2	1.9319	6.1942	6.4775	4.9930	2.6
Del:A118	L1	0.1833	0.5856	0.4376	0.3842	2.1
	L2	0.2006	0.5037	0.5246	0.4926	2.5

*Rate of increase: abundance at week 4/abundance at week 1.

However, if one looks beyond the master sequence and focuses on the other sequence variants, it can be seen that some of them increase in abundance over time while others decrease. Thus, it becomes possible to identify mutations that favor adaptation and others that repress it. By analyzing the behavior of some variants, especially at week 1, it was observed that most of the sequences that are found disappear quite quickly over time, and that this is true for both libraries (Table 1). For example, Insertion C253 (Ins:C253), Ins:G254, Ins:U254, and Ins:A254 have a high abundance in week 1 as compared to week 4. The case of Ins:C253 is particularly interesting since it represents about 20–24% of the sequences of the quasi-species at week 1, that is to say almost the same level as master sequence at week 1, and represents about 0.5% at week 4. Although this proportion is still high at week 4 (corresponding to the fifth most represented sequence), it represents a decrease of 26- or 45-fold depending on the library. Even if the abundance of the other three mutants (Ins:G254, Ins:U254, and Ins:A25) is smaller, the observed decreases remain at the same order of magnitude, specifically, 21–28-fold in L1 and 41–48-fold in L2. Other sequence variants detected at week 1 contain insertions or deletions in the region around positions 250–255 of the PSTVd-RG1 genome, and in all cases, result in a loss of adaptation and/or fitness, indicating that this region is quite sensitive to any changes in the nucleotide sequence (Supplementary Figure 1).

**FIGURE 3:**
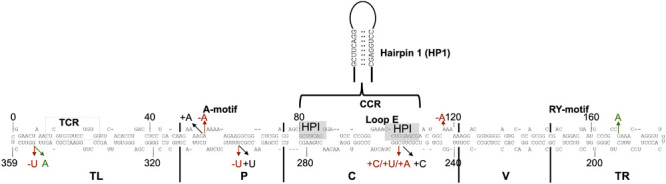
Sequence mutations of the ten most retrieved PLMVd variants. The nucleotide sequence and secondary structure of PSTVd-RG1. The structural/functional domains of PSTVd: TL, P, C, V, and TR are delimited by the vertical solid lines and are named accordingly. The A-motif, Central Conserved Region (CCR), loop E, and RY-motif are indicated. Additionally, the sequence that form the metastable structure hairpin I (HPI) via pairing of distant complementary nucleotides sequences is enlarged. The arrows indicate the positions where mutations occurred. Nucleotide insertion, deletion and substitution are shown in black, red, and green, respectively.

To identify mutations that favor adaptation, attention was focused on the variants most frequently found at week 4 (Table 2). At this stage of the infection, the variants that are found in greater proportion should normally be the best adapted. As previously described, the master sequence occupies the first place with a proportion of 70–72%. In-depth analysis of both the libraries revealed five sequence variants whose frequency increased during the course of infection (Supplementary Figure 1 and Table 2). These are the variants Ins:A55, Deletion A55 (Del:A55), Substitution G168A (Sub:G168A), Del:U296, and Del:A118. For all of these variants, there is an enrichment rate of about 2-fold, regardless of the library. The variant Del:A55 is an exception with a rate of about 95-fold. Unlike the mutations associated with a decreased abundance, the mutations associated with an increased abundance are scattered through the entire genome. All of these mutations of interest are displayed on the genomic structure of PSTVd-RG1 (Figure 3).

### Structural Analysis of the PSTVd Quasi-Species

**FIGURE 4:**
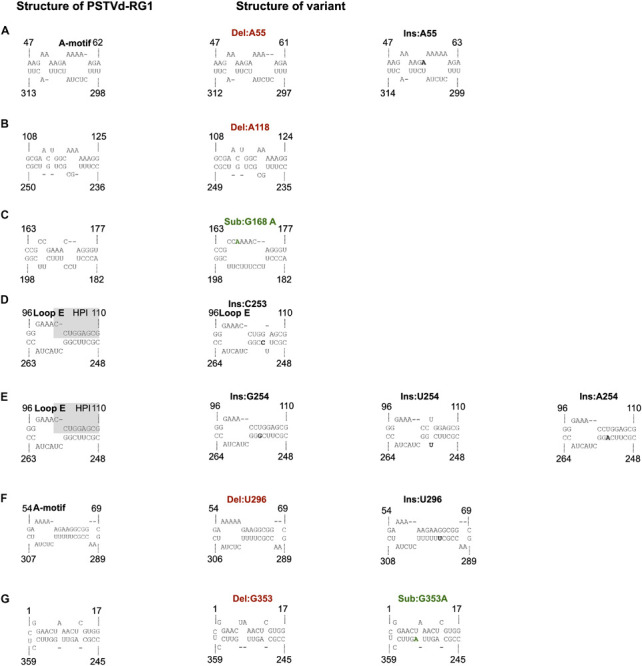
Impact of the mutations on the structure of PSTVd-RG1. **(A)** The deletion or insertion of an adenosine at position 55 results in a decrease and an increase in the size of the A-motif, respectively. A nucleotide in bold indicates an insertion. **(B)** The deletion of the adenosine at position 118 reduces the size of the loop. **(C)** The substitution of adenosine for the guanosine located at position 168 (in green) makes a single larger loop in the TR domain instead of the 2 smaller ones that are present in the wild type. **(D)** The structure of the hairpin I (HPI) is affected by an insertion of one nucleotide (in bold) at position 253. **(E)** The structure of both the hairpin I (HPI) and Loop E are affected by an insertion of one nucleotide (in bold) at position 254. **(F)** The deletion or insertion of a uracil at position 296 resulted in either an increase or a decrease in the size of the A-motif. **(G)** The deletion of the guanine located at position 353 increases the size of the loop in the TL domain, whereas the substitution G353A is a covariation (i.e., G:U base pair becomes an A:U base pair). Nucleotide insertion, deletion and substitution are shown in black, red, and green, respectively.

To understand the importance of the mutations described above, their effects on the structures of the viroids were evaluated. The different sequence variants were submitted to the structure prediction software RNAstructure 6.1 and the resulting structures were compared with that of the PSTVd-RG1 (Figure 4). Variation of the nucleotide sequence by insertion, deletion or substitution resulted in changes in the structures. For example, a change at position 55 resulted in either an increased or a decreased size of the loop located in the upper P domain of PSTVd (Figure 4A). Similarly, the insertion or deletion of adenosine at position 118 resulted in either an increased or a reduced size of the loop located in the central region of PSTVd molecule (Figure 4B). Substitution at position 168 (Sub:G168A) resulted in an increased loop size for the loop located in TR region of the PSTVd (Figure 4C). Insertions at position 253 and 254 affected structure of both the Loop E and HPI of PSTVd, the regions that are known to be involved in viroid infectivity and replication (Figures 4D,E) (Hammond and Owens, 1987; Gas et al., 2007). The deletion of uracil at position 296 introduced a loop in the lower P domain of PSTVd and increased the size of loop A (Figure 4F), while the insertion of uracil resulted in a longer helix and a smaller loop A as compared to PSTVd-RG1. Some mutations altering the nucleotide base-pairing on PSTVd secondary were also observed. For example, the mutation at position 353 from G to A resulted in a more stable helical structure by changing the G:U to A-U base pairs. The deletion of the guanosine residue at position 353 resulted in an increased size of a loop located in the upper TL domain of the PSTVd molecule. These data illustrate the minor structural changes in PSTVd-RG1 that result from the mutations that occur during the course of the infection.

### Potential Impact on the Host Genes Targeted by Viroid Derived Small RNAs

**FIGURE 5:**
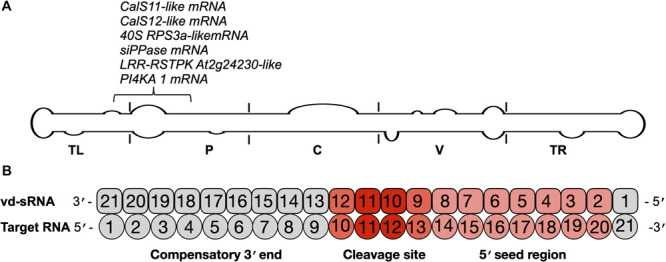
Identification of the mutations on the vd-sRNA known to target host genes. **(A)** The mutations observed in this study were analyzed according to the regions of previously examined vd-sRNAs that are known to downregulate host genes through RNA silencing. These mutations are shown on the potential secondary structure of PSTVd-RG1, and their target genes are indicated. The structural/functional domains of PSTVd are delimited by the vertical solid lines and are named accordingly. Out of all the mutations, the mutation located at position 55 of PSTVd-RG1 showed variation in the vd-sRNA that is known to target the host genes which are involved in defense, development and translation. **(B)** Schematic representation showing the predicted vd-sRNA:target duplex.

RNA silencing is a potent antiviral defense mechanism in eukaryotes directed against invading double-stranded and/or highly structured RNA pathogens such as viruses and viroids. Upon infection, PSTVd-RG1 produces vd-sRNAs of 21–24 nt that are located throughout its genome (Adkar-Purushothama et al., 2017; Adkar-Purushothama and Perreault, 2018). Previously, it has been shown that the vd-sRNAs derived from both the (+) and (−) strands of PSTVd are capable of down-regulating the expression of host mRNAs that are involved in various functions such as defense, development and reproduction through RNA silencing (Eamens et al., 2014; Adkar-Purushothama et al., 2015a, 2017, 2018; Adkar-Purushothama and Perreault, 2018). To examine the presence of mutations in previously verified vd-sRNA that are known to target host mRNAs, all of the PSTVd-sRNAs that are known to target host genes were mapped on the secondary structure of PSTVd-RG1. In the highly expressed PSTVd-RG1 variants, the vd-sRNA sequences that target the *FRIGIDA-like protein 3* (*FRL3*) mRNA, the *serine/threonine-protein kinase At1g01540-like* (*STPK At1g01540-like*), the *chloride channel protein CLC-b-like*, the *putative receptor-like serine/threonine-protein kinase At5g57670* (*RSTPK At5g57670-like*), the *vacuole membrane protein 1-like* (*VMP1-like*) mRNA and the *pentatricopeptide repeat-containing protein At2g17033* (*PPR*) mRNAs were not altered. However, either a deletion or insertion of an adenosine at position 55, which has been observed in some variants, alters the sequence of the vd-sRNA that was shown to induce the RNA silencing of the *CalS11*-*like* mRNA, the *CalS12-like* mRNA, the *40S ribosomal protein S3a-like (40S RPS3a-like)* mRNA, *the putative leucine-rich repeat receptor-like serine/threonine-protein kinase At2g24230-like (LRR-RSTPK At2g24230-like)* mRNA *and* the *phosphatidylinositol 4-kinase alpha* (*PI4KA*) mRNA of tomato (Figure 5A). Since the vd-sRNA derived from this region of PSTVd is involved in targeting different host defense genes, changes in this vd-sRNA could severely affect the fitness and adaptability of the viroid.

For the efficient downregulation of a target mRNA through RNAi-mediated silencing, a strong sequence complementarity is required in both the 5′ seed region of the targeting miRNA and across the cleavage site (Ossowski et al., 2008). To understand the impact of mutants on the host genome, the 21 nt long vd-sRNAs derived from the mutant PSTVd-RG1 sequences were dissected in such a way that the mutant nucleotide within the cleavage site of the vd-sRNA:target duplex (i.e., in either the 10th or 11th position of the vd-sRNA:target duplex; Figure 5B). The resulting vd-sRNAs were then used to interrogate publicly available tomato transcriptome data sets using the WMD3 Web-based tool^2^. The total number of detected targets for each vd-sRNA is given in Table 3. The Gibbs free energy (ΔG), represented as ΔG, for each vd-sRNA:target duplex was calculated using the PairFold software (Andronescu et al., 2003). Although previous *in planta* studies revealed an RNA silencing at ΔG -16.21 (Adkar-Purushothama et al., 2015a), in the present *in silico* analysis, the vd-sRNA:target having a ΔG lower than -20 kcal/mol were grouped and kept for further analysis. The resulting putative target sequences were analyzed by BLAST, and the mRNAs coding for proteins with known functions were sorted (Table 4). The results revealed that the majority of these mutations on PSTVd-RG1 substantially changed the targets of the vd-sRNAs. For instance, the PSTVd-RG1-sRNA derived from the region spanning positions 44–64 (5′-ACAAGAAAAGAAAAAAGAAGG-3′) is known to target the *Pto kinase interactor* 1 (*Pti* 1) which is involved in the *serine-threonine kinase* involved in the hypersensitive response (HR)-mediated signaling cascade, but the mutant is unable to produce vd-sRNA against *Pti* 1 mRNA. Conversely, vd-sRNAs derived from position 108–128 of PSTVd-RG1 had no known target in the tomato genome. That said, the insertion of an adenosine residue at position 118, as was observed in the deep-sequencing data, resulted in the targeting of a probable disease resistance protein. In addition, some mutations favored viroid adaptability by targeting host defense genes, while others showed negative effects on viroid survival by changing the vd-sRNA sequence that was supposed to target the host’s defense genes. Details of the important targets observed in this study, and of the vd-sRNA:target duplexes formed, are shown in Table 4. These data indicate that certain mutations improve viroid accumulation, while others negatively affect its survival.

**TABLE 3:** Vd-sRNAs derived from the master sequence as well as its mutants and the total number of vd-sRNA targets detected in the tomato genome.

**Position on PSTVd-RG1**	**Master sequence vd-sRNA**	**Number targets**	**Mutant**	**Mutant vd-sRNS**	**Number targets**
**MS:55**	ACAAGAAAAG**A**AAAAAGAAGG	17	**Del:A55**	ACAAGAAAAGAAAAAGAAGGC	10
	CAAGAAAAG**A**AAAAAGAAGGC	4	**Ins:-A55**	ACAAGAAAAG**A**AAAAAAGAAG	26
			**Ins:+A55**	CAAGAAAAG**A**AAAAAAGAAGG	10
**MS:118**	GCGAACTGGC**A**AAAAAGGACG	0	**Del:A118**	GCGAACTGGCAAAAAGGACGG	0
	CGAACTGGC**A**AAAAAGGACGG	0	**Ins:-A118**	GCGAACTGGC**A**AAAAAAGGAC	4
			**Ins:+A118**	CGAACTGGC**A**AAAAAAGGACG	2
**MS:168**	AATTCCCGCC**G**AAACAGGGTT	0	**Sub:-G168A**	AATTCCCGCC**A**AAACAGGGTT	0
	ATTCCCGCC**G**AAACAGGGTTT	0	**Sub:+G168A**	ATTCCCGCC**A**AAACAGGGTTT	2
**MS:253**	GCTGTCGCTTCGGCTACTACC	1	**Ins:-C253**	GCTGTCGCTT**C**CGGCTACTAC	0
			**Ins:+C253**	CTGTCGCTT**C**CGGCTACTACC	0
**MS:254**	CTGTCGCTTCGGCTACTACCC	0	**Ins:-A254**	CTGTCGCTTC**A**GGCTACTACC	0
			**Ins:+A254**	TGTCGCTTC**A**GGCTACTACCC	0
			**Ins:-G254**	CTGTCGCTTC**G**GGCTACTACC	0
			**Ins:+G254**	TGTCGCTTC**G**GGCTACTACCC	0
			**Ins:-U254**	CTGTCGCTTC**T**GGCTACTACC	0
			**Ins:+U254**	TGTCGCTTC**T**GGCTACTACCC	0
**MS:296**	CGAGAACCGC**T**TTTTCTCTAT	1	**Del:U296**	CGAGAACCGCTTTTCTCTATC	1
	GAGAACCGC**T**TTTTCTCTATC	5	**Ins:U296**	CGAGAACCGC**T**TTTTTCTCTA	6
			**Ins:U296**	GAGAACCGC**T**TTTTTCTCTAT	4
**MS:353**	AACCGCAGTT**G**GTTCCTCGGA	0	**Del:G353**	AACCGCAGTTGTTCCTCGGAA	1
	ACCGCAGTT**G**GTTCCTCGGAA	0	**Sub:-G353A**	AACCGCAGTT**A**GTTCCTCGGA	0
			**Sub:+G353A**	ACCGCAGTT**A**GTTCCTCGGAA	0

**MS denotes Master sequence; Ins denotes Insertion; Del indicates Deletion; and, Sub represents Substitution. Underlined letters denote the 10th and 11th nucleotides of the vd-sRNA:target RNA duplex. Bold black colored letters indicate the nucleotides on the master sequence that undergoes mutation. Bold red colored letters indicate the nucleotides on the viroid variant that showed mutation when compared to the master sequence. “+” indicates that a mutant nucleotide is located at position 10 of the vd-sRNA:target duplex, while “−” denotes that a mutant nucleotide is located at position 11 of vd-sRNA:target duplex.**

**TABLE 4:** Selected vd-sRNA:mRNA duplexes and the functions of the target host’s genes.

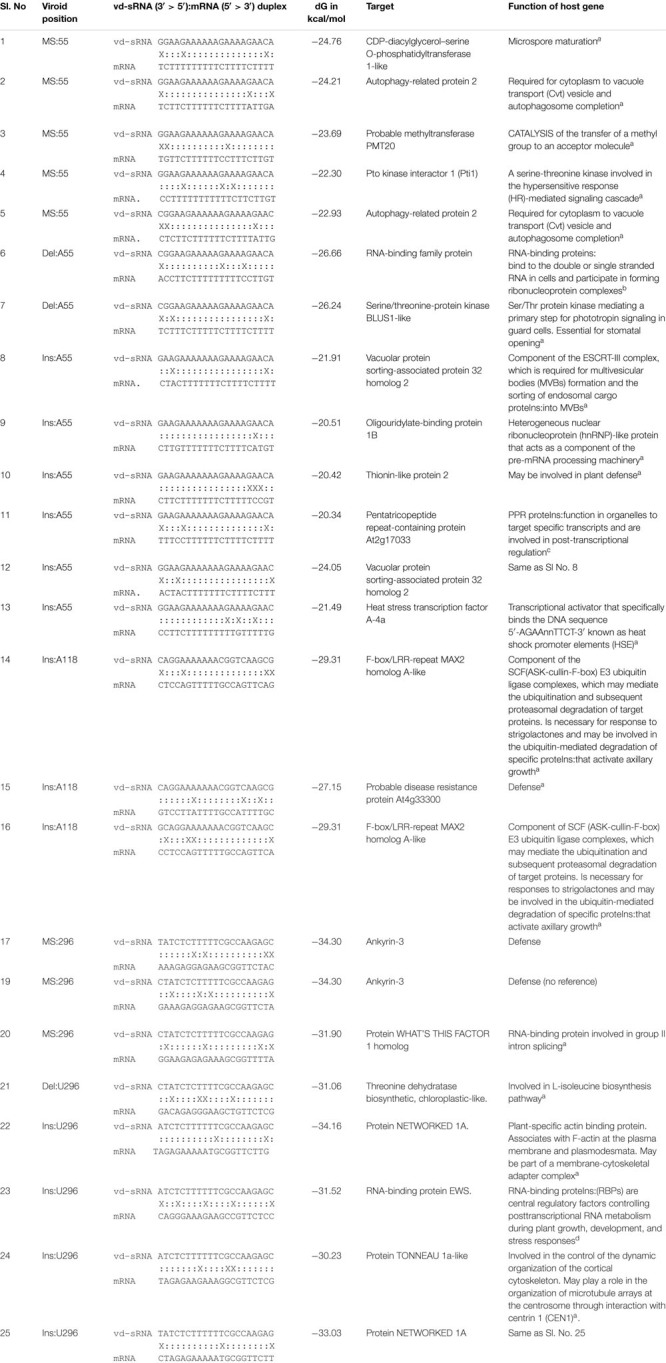

*MS denotes the Master Sequence; Ins denotes Insertion; Del indicates Deletion; and, Sub represents Substitution. ^*a*^https://www.uniprot.org. ^*b*^Dassi (2017). ^*c*^Colcombet et al. (2013). ^*d*^Lee and Kang (2016).*

## Discussion

Upon infection, viroids form a population of sequence variants in their host plant, called quasi-species. This includes the master sequence as well as a certain number of sequence harboring slight differences (Codoñer et al., 2006). Two different forces play crucial roles in defining the viroid quasi-species. Firstly, mutations occur during replication due to the fact that viroids use either the host NEP or the DNA dependent RNA polymerase II for their replication (depending on viroid family), neither of which possess proofreading ability (Gago et al., 2009). The relevant polymerases are indeed not able to perform proofreading, but they have to act even on RNA instead of their natural DNA template, which might increase their error rate while replicating viroid. The second factor that influences viroid quasi-species is host-imposed selection pressure as discussed elsewhere (Tessitori et al., 2013). To date, all of the experiments studying viroid quasi-species have concentrated on analyzing the mutation at a specific point in the infection. While this kind of approach does provide details of viroid quasi-species present at that specific point, it fails to explain the shift in viroid population dynamics during course of the infection as viroid infection follows a S-curve (Figure 2E). This proposes that during the course of infection the viroid titer, as well as the host’s morphological and physiological condition, are constantly changing. In the present study samples were collected from 1st to 4th wpi, which covers the different phases of viroid infection. After verifying the systemic infection of PSTVd in inoculated plants, leaf samples obtained from different plants for each week were subjected to library preparation using two different sets of primers and then subjected to paired end sequencing using the Illumina platform. Detailed analysis of each sequence that was detected at least ten times in a given pool per week showed a maximum of 6.1% divergence from the master sequence at 1 wpi. Except for one particular sequence, both libraries had about a 4.6% difference from master sequence. These data are very similar to the previous mutagenic studies performed on PLMVd, a member of the family, *Avsunviroidae* (Glouzon et al., 2014).

To act as an efficient pathogen, it is crucial for a viroid to adapt itself to the host environment. To understand how quasi-species could help the master sequence to adapt, the ten most abundant sequence variants were further considered, ensuring that the analysis was not biased by technical sequencing errors. As shown in Supplementary Figure 1, of the ten sequence variants, a few were found in both libraries. To increase the confidence in the data analysis, variants recovered from both the L1 and the L2 libraries were analyzed for their adaptability and fitness. It is interesting to note that at 1 wpi the master sequence accounted for 22% of the quasi-species. It then increased in week 2 to as high as 77% before declining slightly. It was also observed that the variants Ins:G254 and Ins:C253, which accounted for more than 20% at week 1 in both libraries, fell below 0.2% at week 2 and then recovered in the later stages of infection (i.e., up to 0.8 at week 4). It should be noted that the mutations at positions 253 and 254 affected sequences that are involved in the formation of HPI and Loop E structures of PSTVd, that are known to involved in viroid infectivity and replication (Figure 3; Hammond and Owens, 1987; Schrader et al., 2003; Gas et al., 2007). This may explain why these sequence variants were not detected after week 1. While this region is quite sensitive to any changes in the nucleotide sequence, deep-sequence data reveals that this is the most favored mutagenic point on PSTVd-RG1. Similar observations were made for other sequence variants; however, the abundance was less than 10% at any given point of infection. For example, sequence variants such as Ins:A55 and Del:U296 showed a gradual increase during the course of infection, while Del:A55 suddenly increased to 4%, from less than 1% during initial stages of infection, at week 4. Although a constant change in sequence variation was observed throughout the infection, the master sequence is the most abundant at any given point of infection, indicating that this is the fittest sequence of the PSTVd quasi-species.

The master sequence is not the only one that consistently increases with time. Some sequence variants showed better adaptability than others by slowly increasing their proportion during the course of infection, consequently they were represented in higher proportions (Table 2). More precisely, the top five variants showed at least a 2-fold enrichment in both libraries. Del:A55 showed a huge enrichment of 95-fold. The adenosine residue located at position 55 is part of a motif-A within the P domain of the PSTVd-RG1 secondary structure. Previous studies have shown that the deletion of this loop resulted in both decreased pathogenicity and trafficking of the viroid molecule as compared to the wild type PSTVd (Zhong et al., 2008).

Previous research demonstrated the involvement of vd-sRNA mediated RNA silencing in viroid pathogenesis irrespective of the viroid family (Adkar-Purushothama and Perreault, 2020). Analysis of the RNA silencing targets of vd-sRNA derived from both the master sequence and the variants provides a potential explanation as to why certain variants are more adaptable and fit. As mentioned earlier, the Del:A55 variant showed highest enrichment over the period of infection. vd-sRNAs derived from this region are probably targeting, in a more efficient way, genes preventing the establishment of infection than those derived from the other variants as well as from the master sequence. At this point, the obvious question is why Del:A55 did not outnumber the master sequence if it targets key genes more efficiently than the latter. There might be several explanations: (i) the master sequence was the one that was initially inoculated, and chances are that its replication is favored as compared to that of the variant Del:A55; (ii) as the deletion of a nucleotide results in a change in the secondary structure, it might also have an effect on the stability of the mutants as well as on their interaction with the host’s components; and, (iii) as infection progresses the host factors are continuously changing and the effect of this on the master sequence as well as on Del:A55 variant is not known. That said, a multi-dimensional approach including, but not limited to, structural analysis, study of the interaction of host components with each variant, biochemical, bioinformatic and biotechnological techniques are required to shed light on this problem.

The data presented here shows that the viroid quasi-species is constantly changing during the course of disease as the disease symptoms progress in the host plant. At the initial stage of infection, a quasi-species of viroid sequences is formed with the master sequence representing no more than 25% of the quasi-species. However, it adapted well, stabilized itself and constituted more than 70% during next stages of disease. In other words, the complexity of the quasi-species decreased from 75 to 30% during the course of infection, indicating the survival of the fittest variants during the course of disease. The data presented here raise many questions, such as what will the quasi-species be if another variant is used for the infection (example, Del:A55), what are the driving forces that stabilize the quasi-species of sequences throughout infection, what features of the viroid make it the most adaptive and fittest variant and what are the host factors involved. These studies require further research in this direction to understand the selection pressures that govern the viroid quasi-species stabilization and their interaction with host components.

Note: When this manuscript was in the final stage of review, deep-sequence analysis of columnea latent viroid, a member of the family *Pospiviroidae*, revealed 79–561 viroid sequence variants, depending on the plant species (Tangkanchanapas et al., 2020).

## Data Availability Statement

The datasets presented in this study can be found in online repositories. The names of the repository/repositories and accession number(s) can be found below: https://www.ncbi.nlm.nih.gov/geo/, GSE147577.

## Author Contributions

CA-P, PB, and J-PP conceived and designed the experiments. CA-P and PB performed the experiments. CA-P and FB analyzed the data. J-PP contributed reagents, materials, and analytical tools. CA-P, FB, and J-PP contributed to the writing of the manuscript.

## Conflict of Interest

The authors declare that the research was conducted in the absence of any commercial or financial relationships that could be construed as a potential conflict of interest.

## Footnotes

1 http://palace.lgfus.ca

2 http://wmd3.weigelworld.org/cgi-bin/webapp.cgi
