# Supplementary material for: Insights Into Potato Spindle Tuber Viroid Quasi-Species From Infection to Disease
Source: Front Microbiol. 2020 Jul 3;11:1235. doi: 10.3389/fmicb.2020.01235 (PMC7349936; doi:10.3389/fmicb.2020.01235)
Supplement: Supplementary file 1 [file Data_Sheet_1.PDF]

Supplementary Table 1. Sequences of the primers used for deep-sequencing.

PRIMER NAME	PRIMER SEQUENCE (5' TO 3')
**F1**	aatgatacggcgaccaccgagatctacactctttccctacacgacgctcttccgatctGAAGCTCCCGAGAACCGC
**R1**	agacgtgtgctcttccgatctAGTTGTTTCCACCGGGTA
**F2**	aatgatacggcgaccaccgagatctacactctttccctacacgacgctcttccgatctCGTGGTTCCTGTGGTTCA
**R2**	agacgtgtgctcttccgatcAGTTTAGTTCCGAGGAAC

Nucleotides shown in small letters are complementary sequences for Illumina index primers and nucleotides shown in capital letters are complementary to PSTVd.
