# Supplementary figures and images for: Insights Into Potato Spindle Tuber Viroid Quasi-Species From Infection to Disease
Source: Front Microbiol. 2020 Jul 3;11:1235. doi: 10.3389/fmicb.2020.01235 (PMC7349936; doi:10.3389/fmicb.2020.01235)

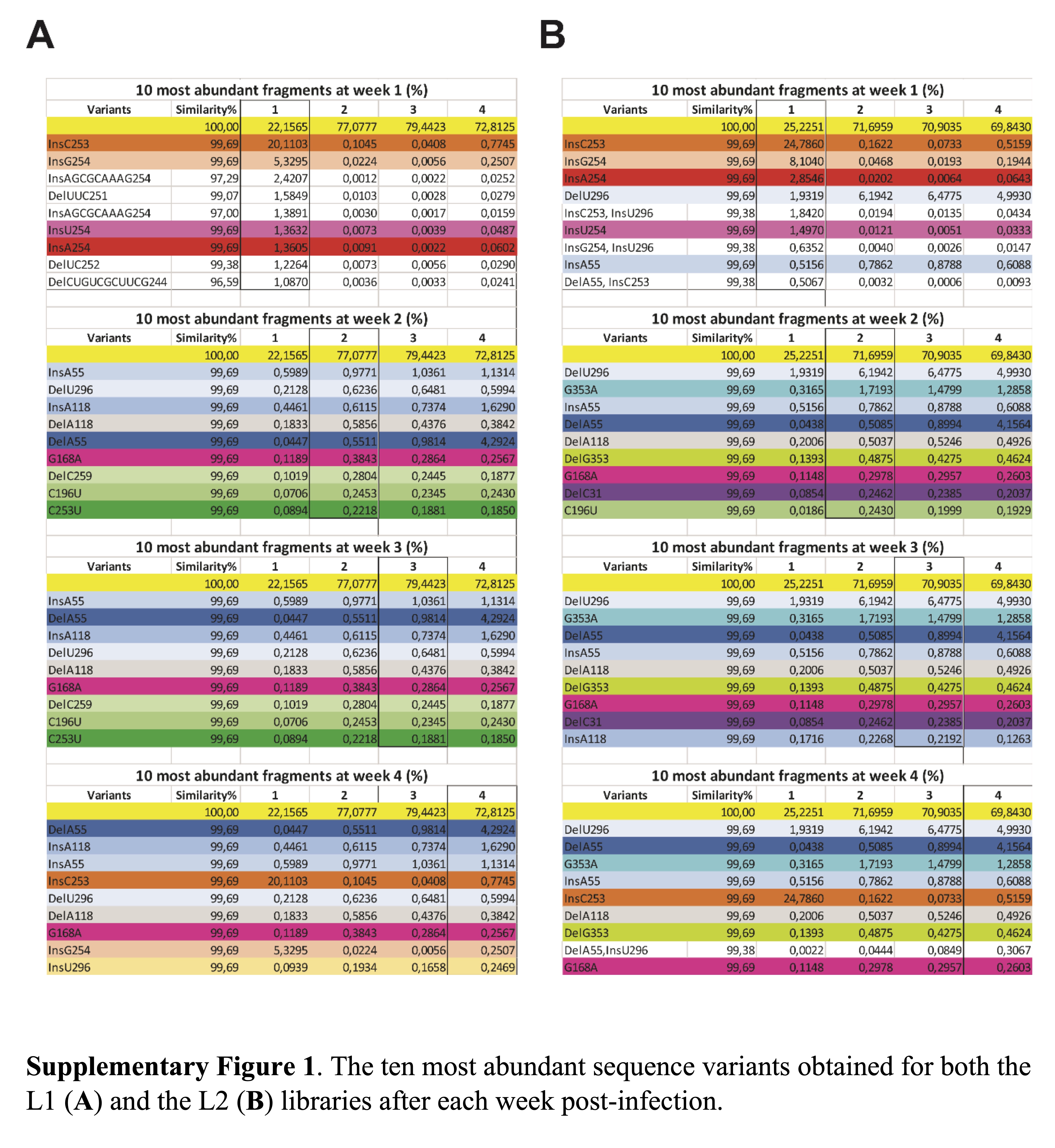

Supplement: Supplementary file 2 [file Image_1.JPEG]
